# Full-Genome Deep Sequencing and Phylogenetic Analysis of Novel Human Betacoronavirus

**DOI:** 10.3201/eid1905.130057

**Published:** 2013-05

**Authors:** Matthew Cotten, Tommy T. Lam, Simon J. Watson, Anne L. Palser, Velislava Petrova, Paul Grant, Oliver G. Pybus, Andrew Rambaut, Yi Guan, Deenan Pillay, Paul Kellam, Eleni Nastouli

**Affiliations:** Wellcome Trust Sanger Institute, Hinxton, UK (M. Cotten, S.J. Watson, A.L. Palser, V. Petrova, P. Kellam);; University of Oxford, Oxford, UK (T.T. Lam, O.G. Pybus);; University College London, London, UK (D. Pillay, P. Kellam); University College London Hospitals,; London (P.Grant, E. Nastouli);; University of Edinburgh, Edinburgh, Scotland, UK (A. Rambaut);; Fogarty International Center–National Institutes for Health, Bethesda, Maryland, USA (A. Rambaut);; The University of Hong Kong, Hong Kong (Y. Guan)

**Keywords:** disease transmission, infectious, coronavirus infections, evolution, molecular, respiratory tract infections, high-throughput nucleotide sequencing, computer-aided design, viruses, zoonoses

## Abstract

A novel betacoronavirus associated with lethal respiratory and renal complications was recently identified in patients from several countries in the Middle East. We report the deep genome sequencing of the virus directly from a patient’s sputum sample. Our high-throughput sequencing yielded a substantial depth of genome sequence assembly and showed the minority viral variants in the specimen. Detailed phylogenetic analysis of the virus genome (England/Qatar/2012) revealed its close relationship to European bat coronaviruses circulating among the bat species of the Vespertilionidae family. Molecular clock analysis showed that the 2 human infections of this betacoronavirus in June 2012 (EMC/2012) and September 2012 (England/Qatar/2012) share a common virus ancestor most likely considerably before early 2012, suggesting the human diversity is the result of multiple zoonotic events.

The ability of coronaviruses (CoVs) to infect multiple species and to rapidly change through recombination presents a continuing human health threat. The epidemic of severe acute respiratory syndrome (SARS) during 2003–2004 during which a CoV transmitted from bats to civet cats and then to humans demonstrated this potential (reviewed in [*1*,*2*]). Recently, a novel human betacoronavirus (betaCoV) was found to be associated with at least 13 human infections, 7 of which were fatal (*3*–*7*). One of the viruses, EMC/2012, has been sequenced, and its sequence similarity to several bat CoVs suggested an animal origin, but a definitive bat species of origin has not yet been identified (*3*). Additional genome sequences from this virus are needed to aid diagnostics, monitor population dynamics, identify the animal source, and characterize mechanisms of pathogenesis. The large size (30,000 nt) and high variability of CoV RNA genomes present a challenge for sequencing.

We describe a strategy for rapidly designing the primers necessary for reverse transcription and cDNA amplification of such diverse RNA viruses and report the full-genome determination of the novel CoV directly from patient sputum using next-generation short-read sequencing. Full genomes from 2 epidemiologically unlinked novel CoV infections separated in time by >2 months were analyzed to gain clues to 2 major questions: what are the precursors of the virus, and how long has the virus been circulating in its current form?

## Materials and Methods

### Primer Design

**Figure 1:**
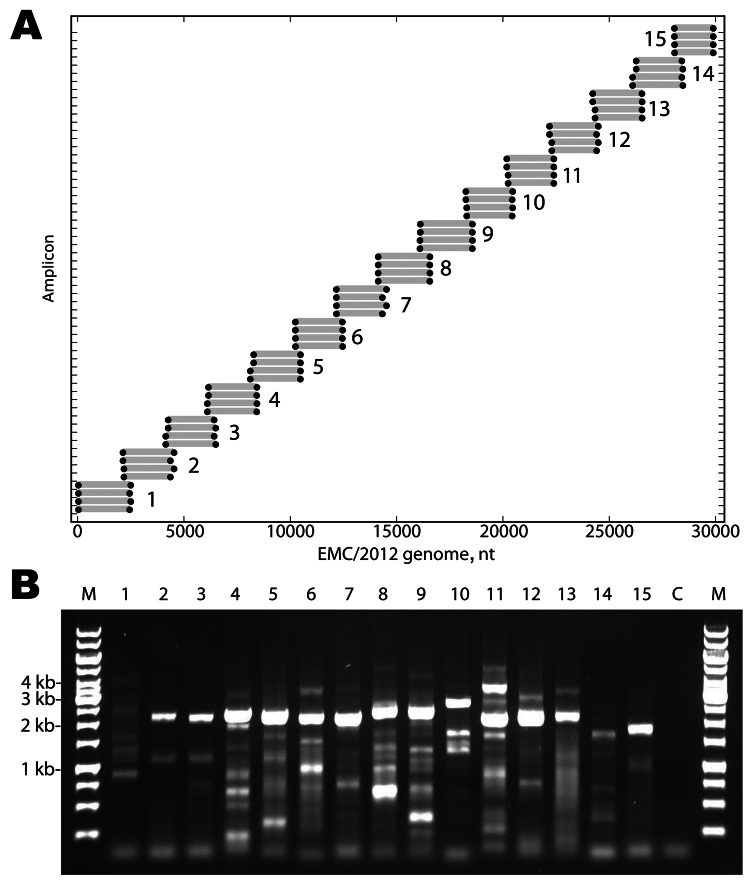
A) Primers designed for reverse transcription and overlapping PCR amplification of the novel coronavirus (CoV). Dots indicate the predicted binding site of each primer along the EMC/2012 genome (x-axis). Gray bars indicate predicted amplicon lengths. Amplicon numbers are indicated beside each set of products. B) PCR products (3 µL of a 25-µL reaction) were resolved by electrophoresis on a 0.6% agarose gel and visualized by ethidium bromide staining. Lane M is the molecular weight marker (sizes indicated at left), Lanes 1–15 show the products of the amplicons depicted in Panel A. Lane C is the reagent PCR control.

Fifteen betaCoV full genomes with the closest homology to EMC/2012 (GenBank accession no. JX869059) were analyzed to identify all possible primer-like sequences (melting temperature 58°C–60°C, guanine plus cytosine content 35%–60%) yielding 357,198 potential primers. The 30-kb JX869059 CoV genome was divided into fifteen 2.5-kb overlapping amplicons, and the 2 highest frequency sequences mapping in the 5′ and 3′ 250 bp of each amplicon were selected. Reverse complements of the 3′ mapping sequences were prepared, resulting in a set of 60 primers for the 15 amplicons (4 primers/amplicon). Three additional primers were added for the extreme termini of the genome. The algorithm also prepared a virtual PCR map of the predicted binding and amplicon size for the primer set on CoV genomes. A map of the primer mapping positions and the predicted PCR products using EMC/2012 as the target is shown in Figure 1, panel A. The primer sequences and details of their use are listed in the Technical Appendix Table.

### Sample History

The patient, originally from Qatar, visited Saudi Arabia, where he became unwell with severe respiratory disease and renal failure and was transferred to a hospital in London, UK (*4*). Viral RNA was extracted from a sputum sample collected on September 19, 2012, sixteen days after symptom onset. Real-time PCR was performed by using an assay targeting the *upE* gene (*6*) and confirmed by using primers and probe targeting the RNA-dependent RNA polymerase (*RdRp*) gene. RdRpTaq1: (5′-GAC TAA TCG CCA GTA CCA TCA G-3′), RdRpTaq2: (5′-GAA CTT TGT AGT ACC AAT GAC GCA-3′), RdRpProbe: (5′-FAM-ATG CTT AAG TCC ATG GCT GCA ACT CGT GGA G–BHQ-3′). The assays gave cycle threshold values of 17.83–18.01 for the *upE* and *RdRp* targets. A 100-μL sample of sputum was lysed with the addition of an equal volume of Qiagen Lysis Buffer AL (QIAGEN, Hilden. Germany). RNA was extracted from 200 µL of this material (final elution volume 60 µL) by using the EZ1 Virus Mini Kit v2.0 and EZ1 instrument (QIAGEN).

### Reverse Transcription and PCR Amplification

Reverse transcription was performed at 50°C for 60 min by using the amplicon reverse primers. PCRs were then performed with forward and reverse primers for each amplicon (15 reactions) for 35 cycles (98°C, 10 sec; 54.5°C, 30 sec; 72°C, 2.5 min) with a final 10-min 72°C extension. A 3-µL aliquot of each reaction was analyzed by electrophoresis, and each reaction showed the expected 2–2.5-kb products (Figure 1, panel B). A detailed protocol is available on request from the authors.

### Sequencing

The PCR products of amplicons were combined into 4 pools of approximately equal molarity and processed into standard Illumina multiplex libraries (Illumina, San Diego, CA, USA) with each pool bearing a unique bar code sequence. Libraries were sequenced by Illumina MiSeq generating 150-bp paired end reads. The resulting Illumina read sets were processed to remove adaptor and primer sequences and quality controlled to ensure a median read Phred quality score of 30 by using QUASR (*8*). The 5′- and 3′-most primers were derived directly from the first 27 nt or last 31 nt of the EMC/2012 genome. These primer sequences were not trimmed from the reads, which might mask sequence difference between EMC/2012 and England/Qatar/2012 at these positions.

### Genome Assembly

Reference-based mapping and de novo assembly methods were applied to the data for assembly into viral genomes. Reference-based mapping was performed against the EMC/2012 genome by using the Burrows-Wheeler Aligner software package [9]). For de novo assembly, maximum contig lengths were obtained by using subsets of 30,000–60,000 reads. Therefore, random subsets of reads were extracted from the readset and assembled by using Velvet version 1.2.07 (*10*,*11*) and VelvetOptimiser (*12*). The de novo assembled sequences were used to confirm the validity of the reference-based sequence and showed that the de novo assembly and the reference-based mapping produced identical sequences, apart from several small gaps near the termini of the de novo assembled sequence (results not shown). The complete genome sequenced here is named England/Qatar/2012 and is available in GenBank (accession no. KC667074).

### Sequence Alignment

The complete England/Qatar/2012 genome was combined with 46 previously published complete genomes of the α-(group 1), β-(group 2), γ-(group 3), and δ-(group 4) CoVs. The betaCoV complete genomes encompassed the a, b, c, and d lineages, as well as the 2 genomes of the novel human betaCoV, EMC/2012 (GenBank accession no. JX869059) and England1 (GenBank accession no. KC164505), the latter from the same patient as studied here. The sequences were aligned by using MUSCLE (*13*) within MEGA5 (*14*). Poorly aligned regions and the genes absent in some CoV genotypes were excluded from the alignment, resulting in a virtual concatenation of the open reading frame (ORF) 1ab, S, E, M, and N genes. A second alignment using a 396-bp region of the *RdRp* was generated similarly. This shorter alignment included strains that are genetically close to England/Qatar/2012 but lack complete genome sequences in GenBank.

### Phylogenetic Methods

Maximum-likelihood (ML) phylogenetic trees were inferred from the sequence alignments by using PhyML version 3.0 (*15*). Phylogenies inferred from nucleotide and amino acid sequences used the general-time reversible and Whelan-and-Goldman substitution models, respectively. A discrete-Γ distribution of 4 rate categories (Γ_4_) was used to model among-site heterogeneity. The robustness of the tree topology was assessed by bootstrap analysis of 1,000 pseudo-replicates of the sequences.

### Molecular Clock Dating

The evolutionary time scale of 2 novel human betaCoVs, EMC/2012 and England/Qatar/2012, was estimated by using a strict clock model under the Bayesian Markov Chain Monte Carlo framework in BEAST version 1.7.4 (*16*). The Hasegawa-Kishino-Yano nucleotide substitution model was used with a discrete-Γ distribution of 4 rate categories (Γ_4_) to enable among-site heterogeneity. A simple constant coalescent prior on the age of the divergence was used. With only 2 samples, estimating a rate of evolution was not possible, so we conditioned our date estimates on a range of fixed rates from 1.0 × 10^−4^ to 5.0 × 10^−3^ substitutions/site/year to enable comparison with plausible values. For each fixed rate value, a total of 10^7^ Bayesian Markov Chain Monte Carlo states were computed, with the first 10% discarded as burn-in.

## Results

### Comparison of England1, England/Qatar/2012, and EMC/2012

The index genome for this virus, EMC/2012, was originally obtained from a Saudi Arabian patient in July 2012 after 6 passages in cell culture (*3*). Our England/Qatar/2012 sequence was derived directly from the sputum of a Qatari patient receiving care in London in September 2012. A consensus genome from the same Qatari patient, but from a lower respiratory tract sample obtained later in the infection, is also available (England1, GenBank accession no. KC164505). The 3 genomes were aligned to determine sequence differences.

**Figure 2:**
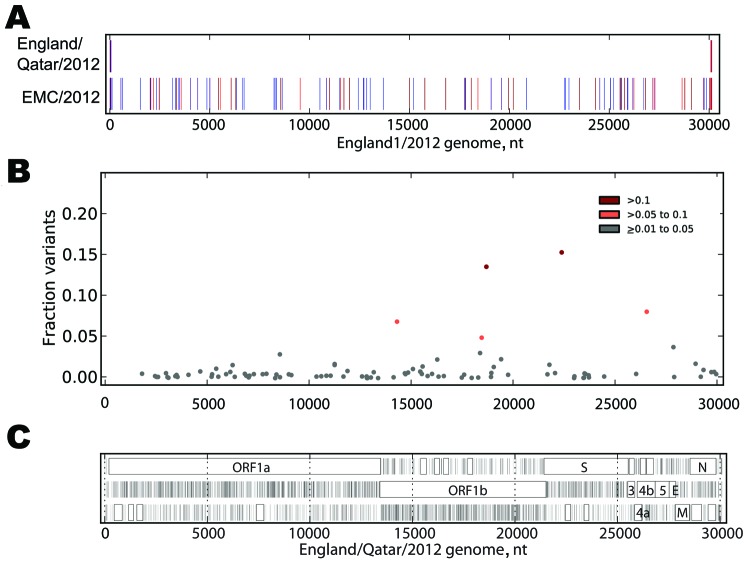
A) Sequence differences among EMC/2012, England/Qatar/2012 and England1. The sequences of the 3 genomes were aligned, and differences between the sequence of England/Qatar/2012 and England1 (upper row) or EMC/2012 and England1 (lower row) were tabulated. The colored vertical ticks indicate nucleotide differences (change to A: red, change to T: dark red, change to G: indigo, change to C: medium blue, gap: gray). B) Non-consensus variants detected in the virus sample. The Illumina readset (Illumina, San Diego, CA, USA) for England/Qatar/2012 was mapped to the England/Qatar/2012 genome. Nucleotide positions showing nucleotides that differed from the consensus were tabulated. Colored dots indicate nucleotide positions with >1%–5% (gray), >5%–10% (orange), and >10% (red) nonconsensus variants. Positions with >5% variation and observed nucleotides are as follows: 14311: T, 92.07; G, 7.81; C, 0.12. 18460: C, 94.03; T, 5.97. 18692: G, 85.35; T, 14.65. 22385: G, 83.59; A, 16.41; C, 0.01. 26554: A, 90.85; G, 9.00; T, 0.15. C) Open reading frame (ORF) analysis of England/Qatar/2012. The positions of stop codons in each of the 3 forward ORFs are indicated by vertical black lines; the presence of ORFs of >75 aa are indicated by a closed box. ORF nomenclature is from Van Boheemen et al. (*3*).

The England/Qatar/2012 and England1 genomes show only nucleotide differences at the genome termini, with the England1 genome lacking 46 nt at the 5′ end and 42 nt at the 3′ end (Figure 2, panel A, Appendix). The England1 genome was generated by using 80 PCR products sequenced by Sanger dideoxy methods (*17*), and the high level of sequence identity between the England/Qatar/2012 and England1 sequences strongly validates the novel rapid sequencing methods described here.

The England/Qatar/2012 consensus genome has 30,021 (99.67%) of 30,119 nucleotides identical to the EMC/2012 genome. In addition to the single nucleotide differences, EMC/2012 shows an insertion of 6 nt starting at position 29639 and a single A insertion at 30661 relative to the England/Qatar/2012 genome. The sequence differences are evenly spread across the genome (Figure 2, panel A, Appendix; Table 1).

**Table 1:** Nucleotide and amino acid differences between novel human betacoronaviruses EMC/2012 and England/Qatar/2012 major ORFs

ORF*	Nucleotide			Amino acid	
	Difference†	Change, %‡		Difference†	Change, %‡§
ORF 3	4	1.28		2	1.92
N	11	0.86		4	0.94
ORF 8b	2	0.59		1	0.88
ORF 4a	3	0.88		1	0.88
NSP13	4	0.24		4	0.71
NSP2	7	0.35		4	0.61
NSP15	3	0.29		2	0.58
ORF 5	3	0.44		1	0.44
NSP3	21	0.37		8	0.42
NSP4	3	0.20		2	0.39
ORF 4b	4	0.46		1	0.34
ORF 1a	45	0.34		14	0.32
S	10	0.24		2	0.15
ORF 1b	15	0.19		3	0.11
E	0	0.00		0	0.00
M	0	0.00		0	0.00
NSP1	2	0.35		0	0.00
NSP5	2	0.22		0	0.00
NSP6	4	0.46		0	0.00
NSP7	1	0.40		0	0.00
NSP8	1	0.17		0	0.00
NSP9	3	0.91		0	0.00
NSP10	1	0.24		0	0.00
NSP11	0	0.00		0	0.00
NSP12	4	0.14		0	0.00
NSP14	3	0.19		0	0.00
NSP16	1	0.11		0	0.00

*ORF nomenclature is from van Boheemen et al. (*3*). ORF, open reading frame; NSP, nonstructural protein. †No. nucleotide or amino acid differences between aligned ORF or their predicted protein products. ‡No. differences (nucleotide or amino acid) divided by the length of the ORF or the predicted protein. §ORFs were sorted by decreasing amino acid percentage change.

Deep sequencing data enable the generation of a consensus sequence from the majority nucleotide at each genome position and the identification of nonconsensus nucleotides at each position. The England/Qatar/2012 sample was sequenced to a high level of coverage (mean coverage = 4,444) compared with the previously reported England1 genome (2–11-fold), enabling insights into the variation consequent on within-host evolution. Setting a conservative estimate for total sequencing errors at 1% enables nucleotide variants present at >1% frequency to be considered true variants in the virus genome (Figure 2, panel B, Appendix). Variation is clearly detected across the virus genome with certain regions, such as nonstructural protein (NSP) NSP3, NSP5, NSP6, NSP12, and NSP14 showing increased levels of nonconsensus nucleotides (Table 2).

**Table 2:** Nonconsensus variants detected in the sequence of a novel human betacoronavirus

Name*	Start†	End	>0.01–0.05‡	>0.05–0.1	>0.1	Total§	Length	%¶#
NSP9	12659	12988	3	0	0	3	329	0.91
NSP10	12989	13408	3	0	0	3	419	0.72
NSP14	18001	19572	6	1	1	8	1571	0.51
NSP6	10937	11812	4	0	0	4	875	0.46
NSP3	2837	8497	25	0	0	25	5660	0.44
NSP12	13432	16206	11	1	0	12	2774	0.43
NSP7	11813	12061	1	0	0	1	248	0.40
NSP5	10019	10936	3	0	0	3	917	0.33
N	28565	29806	4	0	0	4	1241	0.32
S	21455	25516	12	0	1	13	4061	0.32
ORF 4a	25851	26180	1	0	0	1	329	0.30
M	27852	28511	2	0	0	2	659	0.30
ORF 8b	28761	29099	1	0	0	1	338	0.30
NSP13	16307	18000	5	0	0	5	1693	0.30
NSP4	8498	10018	4	0	0	4	1520	0.26
NSP2	857	2836	4	0	0	4	1979	0.20
NSP8	12062	12658	1	0	0	1	596	0.17
ORF 4b	26092	26832	0	1	0	1	740	0.14
NSP15	19573	20601	1	0	0	1	1028	0.10
NSP1	278	856	0	0	0	0	578	0.00
NSP11	13409	13453	0	0	0	0	44	0.00
NSP16	20602	21513	0	0	0	0	911	0.00
ORF 3	25531	25842	0	0	0	0	311	0.00
ORF 5	26839	27513	0	0	0	0	674	0.00
E	27589	27837	0	0	0	0	248	0.00

*ORF nomenclature is from van Boheemen et al. (*3*). ORF, open reading frame; NSP, nonstructural protein. †Nucleotide position in England/Qatar/2012 genome. ‡No. nucleotide positions showing the indicated fraction of nonconsensus nucleotides. Only positions with a minimum of 1,000-fold coverage and a Phred quality score of 30 were included. §No. nucleotide positions with >0.01 (1%) nonconsensus nucleotides. ¶Total no. positions with variation divided by length of ORF) × 100. #ORFs were sorted by decreasing amino acid percentage change.

The predicted ORFs in England/Qatar/2012 are shown in Figure 2, panel C, Appendix. The ORF pattern is identical to EMC/2012, with 1 exception: England/Qatar/2012 has a G at position 27162 and a longer ORF 5, whereas EMC/2012 has an A (and the predicted ORF 5 is truncated). Van Boheemen et al. (*3*) had commented on mixed nucleotides (A–G) observed at this position. In addition, EMC/2012 had variation at position 11623 (U or G), whereas the England/Qatar/2012 genome has a U at this position.

An assessment of specific ORFs of the 2 viral genomes was performed because evolution of specific proteins is a key determinant of host range and defines cross-species transmission events that may be recent for this virus. As expected from the overall close homology of the 2 viruses, several ORFs show little or no change between the 2 genomes (Table 1). However, several ORFs show higher amino acid differences, including the nucleocapsid ORF N, and ORFs 3, 4a, and 8b (Table 1).

Of the 16 predicted nonstructural proteins encoded by ORF 1a and ORF 1b, 11 show no change at the amino acid level (Table 1), whereas 5 (NSP2, NSP3, NSP4, NSP13, and NSP15) show >0.3% difference (Table 1). As more sequences become available from this virus, such comparisons will yield clues about the adaptation to humans.

### Close Phylogenetic Relationship with European Bat CoVs

**Figure 3:**
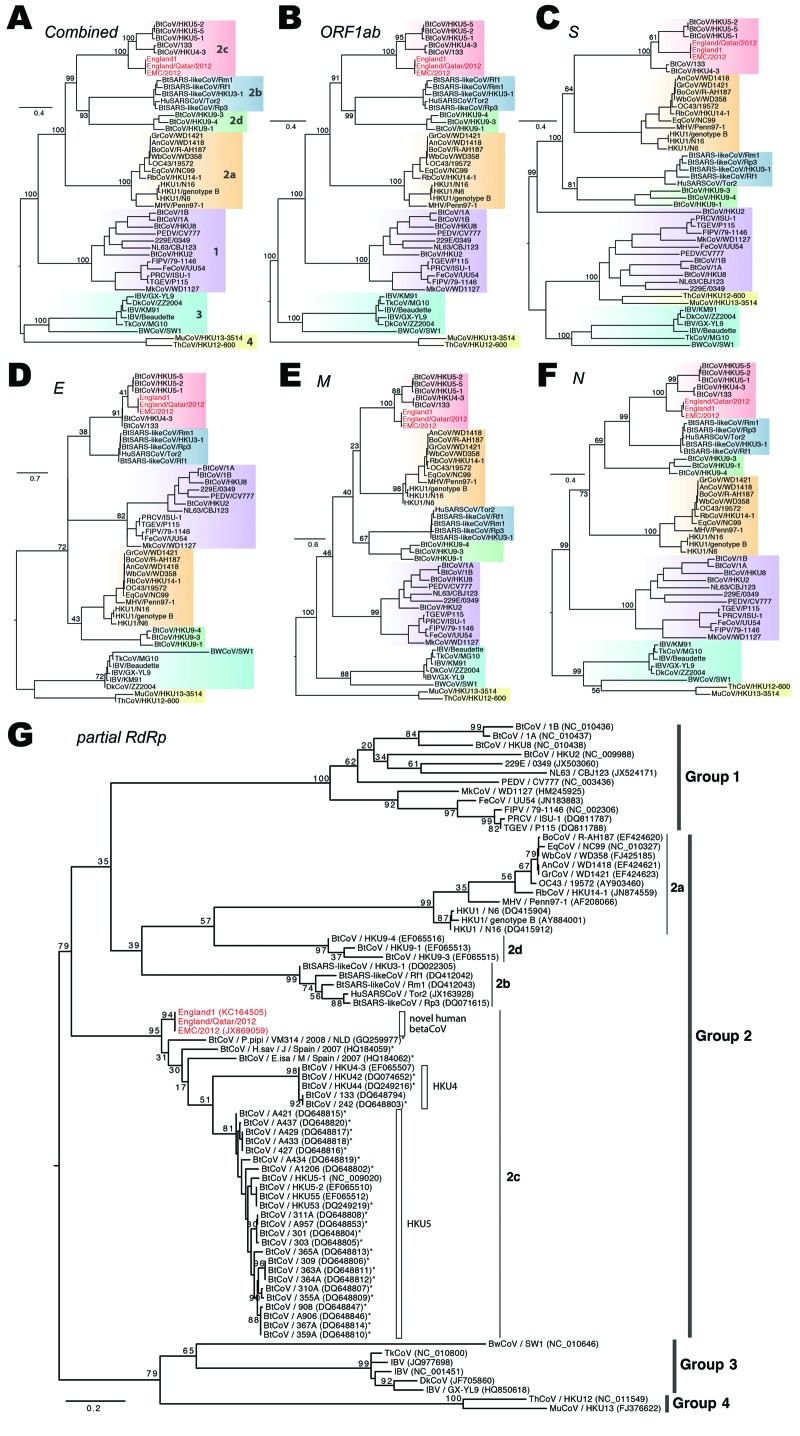
Phylogenetic analyses of coronaviruses. A–F) Maximum-likelihood phylogenies of combined and each individual open reading frame (ORF), including ORF 1ab, S, E, M, and N. Previously defined viral lineages (group 1, 2a, 2b, 2c, 2d, 3, and 4) are highlighted by color blocks and described in (A). G) Phylogenetic analyses on the partial RNA-dependent RNA polymerase sequence region (396 bp) of coronaviruses (CoVs). Partial gene sequences from other CoVs that are closely related to the novel human betaCoVs are included and marked with asterisks. Bootstrap analysis of 1,000 replicates was performed for each phylogeny. The novel human betaCoVs studied here are shown in red. Scale bar indicates nucleotide substitutions per site.

A ML phylogenetic tree inferred from the whole genome alignment indicated that the 3 novel human betaCoVs sequences (England1, England/Qatar/2012, and EMC/2012) clustered closely, forming a monophyletic lineage that falls into group 2c (Figure 3, panel A, Appendix,). This novel human betaCoV lineage shares common ancestries with other group 2c bat CoV variants, including HKU4 and HKU5 strains isolated in southern People’s Republic of China (Figure 3, panel A, Appendix); however, the genetic distances between them remain substantial (≈70% similarity in nucleotide level). The novel human betaCoVs have even less similarity with group 2a, 2b, and 2d CoVs (<60% nt). Phylogenies constructed from individual ORFs including ORF 1ab, S, E, M, and N demonstrated a largely consistent phylogenetic position of the novel human betaCoVs, except for a small discordance in the order of branching between novel human betaCoV, HKU4, and HKU5 lineages (Figure 3, panels B–F, Appendix). Such phylogenetic incongruence could result from several possible evolutionary features of the virus, including evolutionary rate variation, homoplasy, and recombination, as well as from uncertainty in the alignment of distant sequences. Additional related sequences are needed to clarify this issue.

Because more CoV strains are available in GenBank for partial genomic sequences, we performed extensive BLAST searches (www.ncbi.nlm.nih.gov/blast) and identified several additional European bat CoV sequences sharing higher nucleotide sequence similarity (82.0%–87.7%) with the novel human betaCoVs. Because only partial *RdRp* sequences were available for these European bat CoVs, another ML phylogeny was constructed from this short region to examine their evolutionary relationships (Figure 3, panel G, Appendix). As noted, P.pipi/VM314/2008/NLD (GenBank accession no. GQ259977) identified in a *Pipistrellus pipistrellus* bat from the Netherlands (*18*) shows the closest sequence similarity (87.7%) and phylogenetic relationship to the novel human betaCoVs. In addition, 2 CoV sequences, H.sav/J/Spain/2007 (GenBank accession no. HQ184059; from *Hypsugo savii*, also known as *P. savii*) and E.isa/M/Spain/2007 (HQ184062; from *Eptesicus isabellinus*), obtained from bats from Spain (*19*) are also closely related to the novel human betaCoVs (Figure 3, panel G, Appendix).

### Timing the Origin of Zoonosis

**Figure 4:**
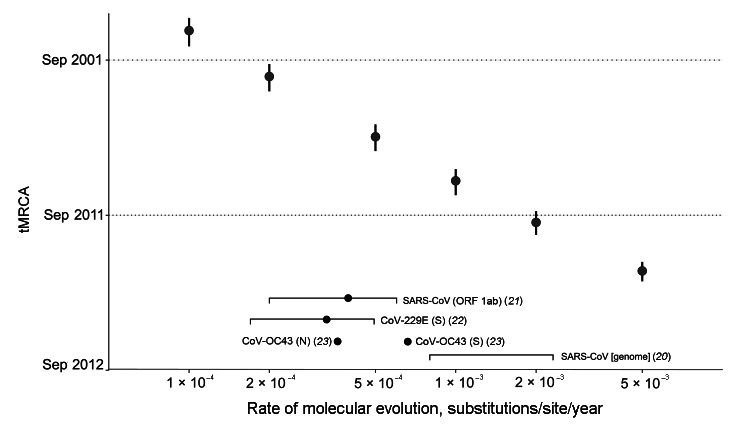
tMRCA analysis across a range of fixed evolutionary rates. The tMRCA of EMC/2012 and England/Qatar/2012 estimated from fixing a range of genomic evolutionary rates (1 × 10^−4^, 2 × 10^−4^, 5 × 10^−4^, 1 × 10^−3^, 2 × 10^−3^, and 5 × 10^−3^ substitutions/site/year) are shown in data points with vertical error bars (95% highest posterior density). Evolutionary rate estimates of human CoV genome and genes in the literature are indicated at the bottom of the plot (mean or point estimate as a dot, 95% CIs of estimate as a square bracket). tMRCA, time to the most recent common ancestor; CoV, coronavirus; SARS, severe acute respiratory syndrome; ORF, open reading frame.

The time to the most recent common ancestor (tMRCA) of EMC/2012 (isolated June 13, 2012) and England/Qatar/2012 (isolated September 19, 2012) was estimated with a strict molecular clock model. A plot of estimated tMRCA of the 2 human strains as a function of the fixed rate of molecular evolution shows that with the fastest measured rate of human CoVs (2 × 10^−3^ substitutions/site/year for SARS-CoV [*20*]) the tMRCA (i.e., October 2011; 95% highest posterior density: August–December 2011) is older than the start of 2012, but with slower rates it is calculated to be much older (Figure 4 [*21**–**23*]). However, for evolutionary rates as high as those of human influenza A virus and HIV (e.g., 3–5 × 10^−3^ substitutions/site/year), the estimated tMRCA becomes compatible with the earliest disease report in Jordan in April 2012 (*7*).

## Discussion

The reports of several human infections by similar strains of a novel betaCoV have raised global concern about a new SARS-like outbreak. Such worry is not misplaced, particularly when little is known about the origin and transmissibility of the virus and the natural history of the disease it causes. In this study, we presented a rapid deep sequencing method to obtain the complete genome sequence of the novel human betaCoV and its minor variants from a patient’s sputum sample, which provides a useful tool to study the origin, evolution, and detection of this novel CoV. Our detailed phylogenetic analyses of the viral genomes also provide additional clues to the emergence and evolution of this virus.

Mammalian zoonoses are often identified as the origin of new human CoV infections including OC43, NL63, and SARS-CoVs (*24**–**27*). In particular, bats, which maintain the largest diversity of CoVs (*28*), are thought to be a natural reservoir of the virus and thus a probable origin of the novel human betaCoV studied here. Indeed, the initial genetic study of the novel human betaCoV demonstrated its phylogenetic relatedness to the HKU5 and HKU4 bat CoVs in southern China (*5*). van Boheemen et al. found a bat CoV sequence from the Netherlands that clusters closely with EMC/2012 (*3*). In addition, most bat CoVs in the group 2c lineage, including the sequence from the Netherlands, were isolated from bat species of the Vespertilionidae family, whereas the SARS-like CoVs in group 2b lineage were mainly from bats from the Rhinolophidae family (*29*). In this study, we showed that 2 other CoVs from bats from Spain of Vespertilionidae again are also clustered near the novel human betaCoVs (Figure 3, panel G, Appendix), which further supports a European Vespertilionidae bat ancestry for this virus. However, such a genealogic link may be indirect and distant because a similar hypothesis for SARS-CoV has been under dispute: whether the CoV jumped from bats directly to humans or through civets or even through some other animals as intermediate hosts (*30*). Therefore, like SARS-CoV, the high dissimilarity between the bat CoVs and novel human betaCoVs makes it difficult to rule out the presence of other intermediate hosts transferring the virus from bats to humans and to confirm the geographic origin of the direct predecessor. With the current data, Europe and the Middle East would be plausible regions for more intensive pilot surveillance studies on bats and other animal reservoirs for this virus.

In the interest of public health, it is critical to determine whether these CoV infections in humans are the consequence of a single zoonotic event followed by ongoing human-to-human transmissions or whether the 3 geographic sites of infection (Jordan, Saudi Arabia, and Qatar) represent independent transmissions from a common nonhuman reservoir. The large genetic diversity of CoV maintained in animal reservoirs suggests that viruses that independently moved to humans from animals at different times and places are likely to be reasonably dissimilar in their genomes, possibly making the multiple transmission events model less likely. Further information is needed to confirm this point because the currently available data are limited. If we calibrate our molecular clock analysis using the evolutionary rate of Zhao et al. (*20*) estimated for SARS-CoV, we dated the tMRCA of EMC/2012 and England/Qatar/2012 viruses to early 2011. Therefore, if both sequenced viruses and the other cases descended from a single zoonotic event, then this tMRCA suggests that the novel virus has been circulating in human population for >1 year without detection and would suggest most infections were mild or asymptomatic. The rate would have to be considerably faster, of a magnitude observed for human influenza A virus, for the tMRCA to be compatible with the earliest known cases in April 2012. Perhaps more probable, therefore, is that the 13 known cases of this disease represent >1 independent zoonotic transmission from an unknown source. Viral sequence data from other patients infected with this novel human betaCoV will help to more accurately estimate the estimate a genomic evolutionary rate specific to this virus, which will then yield a tMRCA estimate closer to the actual time.

A major determinant of SARS pathology is the distribution of the host receptor that the virus has evolved to use for entry. A detailed analysis of EMC/2012 receptor use in cell culture (*31*) revealed that EMC/2012 was capable of infecting a range of mammalian cells, including human, pig, and bat cells, and the virus entry was independent of the ACE2 receptor. Comparing the S genes of England/Qatar/2012 predicts only 2 aa changes (in a 1,367-residue protein). Thus, this broad infection capability of EMC/2012 is likely to be valid for England/Qatar/2012 and would imply a higher possibility for the existence of other intermediate hosts transferring the virus from bats to humans.

Precise identification of the origin of this virus, defining its mode of evolution, and determining the mechanisms of viral pathogenesis will require full-genome sequences from all cases of human infection and substantially more sampling and sequencing from Vespertilionidae bats and other related animals. The sequencing method reported here markedly shortens the time required to process the clinical sample to genome assembly to 1 week and will provide a useful tool to study this novel virus.

The current number of confirmed novel CoV cases is 13, including 7 deaths (www.who.int/csr/don/2013_02_21/en/index.html). A third novel CoV genome sequence was posted on the Health Protection Agency website on February 18, 2013 (www.hpa.org.uk/webw/HPAweb&HPAwebStandard/HPAweb_C/1317136246479). An analysis of the novel CoV genomes from the 3 dates suggests an evolution rate of 4 × 4e^−4^, which would give a tMRCA of early 2009. The analysis is available online at http://epidemic.bio.ed.ac.uk/coronavirus_analysis.

Technical Appendix Primer sequences and details of their use.
