# Supplementary material for: Full-Genome Deep Sequencing and Phylogenetic Analysis of Novel Human Betacoronavirus
Source: Emerg Infect Dis. 2013 May;19(5):736–42. doi: 10.3201/eid1905.130057 (PMC3647518; doi:10.3201/eid1905.130057)
Supplement: Technical Appendix — Primer sequences and details of their use. [file 13-0057-Techapp-s1.pdf]

Article DOI: http://dx.doi.org/10.3201/eid1905.130057

Full-Genome Deep Sequencing and Phylogenetic Analysis of Novel Human Betacoronavirus

Technical Appendix

Technical Appendix Table. Primer sequences and details of their use

Name	Sequence (5′→3′)	Amplicon	Direction
FEP_1	GATTTAAGTGAATAGCTTGGCTATCTC	1	Forward
FCO_TM58_20559	TCTCTTGCAGAACTTTGATTTTAACGA	1	Forward
FCO_TM58_25382	CGTTCTCTTGCAGAACTTTGATTTTAA	1	Forward
FCO_TM58_67791	CCCAGGAGACCTTTGTATGCAAA	1	Reverse
FCO_TM58_70342	GGAATATTAGAGACTCCCTGCCG	1	Reverse
FCO_TM58_66908	ATAAGACTGGCGACTTTATGTCTACAA	2	Forward
FCO_TM58_67327	GATCTTTCAGTAGCTTCTACCTATTTT	2	Forward
FCO_TM58_66922	TATCCCAAAGGCATAGATATAATACCA	2	Reverse
FCO_TM58_68466	TGTAACCACCATTAGTGCGGAC	2	Reverse
FCO_TM58_70157	CGCAATACGTAAAGCTAAAGATTATGG	3	Forward
FCO_TM58_71081	AGTTGACGGTGTGCAATATTATTGC	3	Forward
FCO_TM58_67825	AATGAAGCCCTAAATAGTAACTTCACT	3	Reverse
FCO_TM58_73024	CAACGGTGTGCAATCTAAGCAT	3	Reverse
FCO_TM58_68971	AGTTTGCGTCAAATTTTTGACGTAG	4	Forward
FCO_TM58_70053	ACACCTTTGAGTGTGGAGTCTAC	4	Forward
FCO_TM58_67849	CGGAAAGCTAAATTACACTTACGGC	4	Reverse
FCO_TM58_68526	AGGTGGTTAACCGGAAAGCTAAA	4	Reverse
FCO_TM58_65414	GGTGTGGAGTCTGATGTTGAGA	5	Forward
FCO_TM58_67159	TTGTAATGCGGCTTCAGTTAACC	5	Forward
FCO_TM58_21338	TAACCAACACTACCACAAGAACCA	5	Reverse
FCO_TM58_33984	GTGTAACCAACACTACCACAAGAAC	5	Reverse
FCO_TM58_67793	GCAAACTTGCGTGTTGTTGG	6	Forward
FCO_TM58_71809	CTCCAGCAAACTTGCGTGTT	6	Forward
FCO_TM58_67887	GAATTACAACGCGAAGTTTATTTGAAG	6	Reverse
FCO_TM58_68442	GAAGTTTATTTGAAGCACACAGTGG	6	Reverse
FCO_TM58_2977	AAGGCTTTGCAGAAGGCTGTTA	7	Forward
FCO_TM58_3105	TCTTAAGGCTTTGCAGAAGGCT	7	Forward
FCO_TM58_20080	CACACAACGGTCATCAGTACA	7	Reverse
FCO_TM58_68115	TCATCAACTCCTTAAGAGAGAGCCTAT	7	Reverse
FCO_TM58_20472	ATGCCTGTGCTCTCAATGACC	8	Forward
FCO_TM58_57818	TGCCTGTGCTCTCAATGACC	8	Forward
FCO_TM58_8314	CACCACTTTCAGTCCAGTCACA	8	Reverse
FCO_TM58_34102	TGTAATCACCACTTTCAGTCCAGT	8	Reverse
FCO_TM58_18395	GGACACATGCTTGACAGTTATTCTG	9	Forward
FCO_TM58_18846	TTATTCTGTCATGCTATGTGGTGATAA	9	Forward
FCO_TM58_965	AGCCCAACAAACAAACGTACAG	9	Reverse
FCO_TM58_981	CATGAGCCCAACAAACAAACGTA	9	Reverse
FCO_TM58_26334	GCATGTGGCACCAATGTG	10	Forward
FCO_TM58_27585	GGATAGGCTTCGATGTTGAGGG	10	Forward
FCO_TM58_67807	CCGCCTTAAAAGCCGCTG	10	Reverse
FCO_TM58_69145	AGCTTTAAATCTATAACAGAACACACC	10	Reverse
FCO_TM58_67761	GGAAAACTATGCTTTTGAGCACGTA	11	Forward
FCO_TM58_72684	TTCTGACATGGAGAAAGACTTTCTATC	11	Forward
FCO_TM58_28953	GAAGGCAGCCCAAGCTTTTC	11	Reverse
FCO_TM58_34878	TAGAAGGCAGCCCAAGCTTTT	11	Reverse
FCO_TM58_67088	CCACCTTGCCTGTTTATGATACTATTA	12	Forward
FCO_TM58_67744	TACCGAAGATGAGATTTTAGAGTGGTT	12	Forward
FCO_TM58_67030	CAACACCGTTTAACCTATAAAAGATAC	12	Reverse
FCO_TM58_72579	CTGTTTGCATAGCTCCCAGAG	12	Reverse
FCO_TM58_66781	TGGACTGCTGGCTTATCCTC	13	Forward
FCO_TM58_69085	TTATTTGTGCTCAATATGTGGCTGG	13	Forward
FCO_TM58_66820	GCTTAAATCTATGTATGTTAGCACAGT	13	Reverse
FCO_TM58_67938	TATGTATGTTAGCACAGTAGATCTCTG	13	Reverse
FCO_TM58_68075	GAATCCCTGATGGATGTTCCCTC	14	Forward
FCO_TM58_68828	GTTACATTTTCAGACCCCAACATGT	14	Forward
FCO_TM58_69858	TGTAATTACCTGCCTTATATCTATGGT	14	Reverse

Name	Sequence (5′→3′)	Amplicon	Direction
FCO_TM58_70813	TTCCGTAGCTTTGCCGCTT	14	Reverse
FCO_TM58_8770	AGCTTCCCAGATAATCTCTGGC	15	Forward
FCO_TM58_20019	CAATTGATCTAGCTTCCCAGATAATCT	15	Forward
FCO_TM58_879	CATTCTGTGCAAGAGTGGACAA	15	Reverse
FCO_TM58_1015	CCATTCTGTGCAAGAGTGGACA	15	Reverse
FEP_2	TTTTTTTTTTTTGCAAATCATCTAATTAGCC	15	Reverse
FEP_3	GCAAATCATCTAATTAGCCTAATCTAATTG	15	Reverse
